# Carboxymethyl Cellulose (CMC) as a Template for Laccase-Assisted Oxidation of Aniline

**DOI:** 10.3389/fbioe.2020.00438

**Published:** 2020-05-14

**Authors:** Euijin Shim, Jennifer Noro, Artur Cavaco-Paulo, Hye Rim Kim, Carla Silva

**Affiliations:** ^1^Department of Clothing and Textiles, Sookmyung Women’s University, Seoul, South Korea; ^2^Centre of Biological Engineering, University of Minho, Campus of Gualtar, Braga, Portugal; ^3^International Joint Research Laboratory for Textile and Fiber Bioprocesses, Jiangnan University, Wuxi, China

**Keywords:** aniline, laccase, oxidation, template, carboxymethyl cellulose

## Abstract

Polyaniline (PANi) is a conducting polymer which has been subject of intensive research on the exploitation of new products and applications. The main aim of the work is the development of a conductive bacterial cellulose (BC)-based material by enzymatic-assisted polymerization of aniline. For this, we study the role of carboxymethyl cellulose (CMC) as a template for the *in situ* polymerization of aniline. Bacterial cellulose was used as the supporting material for the entrapment of CMC and for the *in situ* oxidation reactions. The amount of CMC entrapped inside BC was optimized as well as the conditions for laccase-assisted oxidation of aniline. The new oligomers were evaluated by spectrometric techniques, namely ^1^H NMR and MALDI-TOF, and the functionalized BC surfaces were analyzed by thermogravimetric analysis (TGA), Fourier-transform infrared spectroscopy (FTIR), X-ray diffraction (XRD), scanning electron microscope (SEM), and reflectance spectrophotometry. The conductivity of the developed materials was evaluated using the four-probe methodology. The oligomers obtained after reaction in the presence of CMC as template display a similar structure as when the reaction is conducted only in BC. Though, after oxidation in the presence of this template, the amount of oligomers entrapped inside BC/CMC is considerably higher conferring to the material greater electrical conductivity and coloration. The use of CMC as a template for aniline oxidation on BC seems to be a promising and cheap strategy to improve the yield of functionalization and increment the properties of the materials, namely electrical conductivity and coloration.

## Introduction

Conductive materials have been gaining scientific attention due to the increasing need for new technologies for the exploitation of electronic sensor devices, energy-storage, and intelligent clothing (Sapurina and Shishov, 2012). Bacterial cellulose (BC) has been used to develop composites containing a conductive polymer, such as polyaniline (PANi), polypyrrole and polythiophene, and others (Hu et al., 2011; Marins et al., 2011; Shim et al., 2019b). The use of templates, like sulfonated polystyrene as sodium salt (SPS), the calcium salt of ligninosulfonate, micelles composed by sodium dodecylbenzenesulfonate (SDBS), or vesicles made-up of sodium bis(2-ethylhexyl)sulfosuccinate (AOT) have been described as favoring the polymerization of aniline (Walde et al., 2019). These molecules are composed by sulfonate groups and, in oxidation conditions, aniline is oxidized to a conductive product, emeraldine salt (Walde et al., 2019). The templates, due to a localization of the reaction in their vicinity, direct the regioselectivity of the monomer coupling reaction, favoring *para-*over *ortho-*coupling of oxidized aniline. These compounds have dopant action (counter ions), which balance the positive charge on the PANi, thereby stabilizing the PANi-Emeraldine salt structure, crucial for electrical conductivity. Together with templates, the laccase/O_2_- assisted polymerization of aniline has been studied as an environmentally friendly route to produce conductive PANi (Streltsov et al., 2009). Laccase has been applied for the aniline polymerization *in situ* inside BC nano fibers under mild conditions replacing the chemical oxidants that are normally used, such as ammonium peroxydisulfate, potassium dichromate or ferric chloride (Shim et al., 2019a). The use of templates is crucial to reduce the undesired coupling reactions, as side-chain branching, and to ensure the polymerization of linear head-to-tail aniline (De Salas et al., 2016). The template works by forming polymer–polymer complexes which are stabilized via non-covalent binding forces among hydrogen bonds, electrostatic and hydrophobic interactions during polymerization (Kim et al., 2004; Yue et al., 2016).

Carboxymethyl cellulose (CMC), a soluble derivative of cellulose, is an example of an efficient template for aniline polymerization. It adsorbs irreversibly to cellulose fibers under specific conditions increasing their negative charge (Peng et al., 2012; Fu et al., 2015; Wang et al., 2016). CMC contains –COO^–^ groups which supply anionic locations to react with electropositive molecules (positively charged cations) via electrostatic interactions, favoring the polymerization of aniline (Fu et al., 2015). BC has considerable amount of hydroxyl groups which, due to their high reactivity, can be easily modified. However, the reactivity of the hydroxyl groups can be restricted by intramolecular and intermolecular hydrogen bonds during polymerization events (Kim et al., 2004; Yue et al., 2016). When CMC is introduced inside BC, the –COO^–^ groups of CMC can form intermolecular interactions with the hydroxyl groups of BC. The loss of some –OH groups by BC, able to interact with other compounds, can be counterbalanced by the presence of CMC, which is composed by hydroxyl and carboxylate groups.

In this work, we developed conductive BC composites by entrapping CMC inside BC membranes followed by the *in situ* aniline polymerization by laccase. The CMC was entrapped inside BC to serve as template for polymerization and laccase was used as reaction catalyst. Potassium hexacyanoferrate (II) (KHCF), a radical initiator, and bis(2-ethylhexyl) sulfosuccinate sodium salt (AOT), a surfactant, were used as additives for aniline oxidation. The role of CMC on aniline polymerization was evaluated through the quantification of the amount of polymer formed. The polymerization of aniline was evaluated by UV/Visible spectroscopy and the new oligomers were characterized by spectrometric techniques, namely ^1^H NMR and MALDI-TOF. BC/PANi and BC/CMC/PANi composites were monitored by FTIR, SEM, TGA, and XRD analysis. The conductivity of the developed materials was assessed through the four-probe method and the color of BC samples was evaluated spectrophotometrically.

## Experimental

### Materials

Glucose (Duksan Pure Chemicals Co., Seoul, South Korea) was used as carbon source. A mixture of yeast extract (Becton, Dickinson and Company, Sparks, Unitesd States) and peptone (Becton, Dickinson, Sparks, United States) were used as nitrogen sources. The following chemicals were used without further purification: acetic acid (Duksan Pure Chemicals Co., Seoul, South Korea), sodium acetate (Sigma, Saint Louis, MO, United States), hydrogen peroxide (Duksan Pure Chemicals Co., South Korea), aniline (Junsei Chemical Co. Ltd., Tokyo, Japan), KHCF (Fisher Scientific, Loughborough, United Kingdom), AOT (Tokyo Chemical Industry Co., Tokyo, Japan). Laccase (EC 1.10.3.2.) from *Myceliophthora thermophila* was obtained from Novozymes (Bagsvaerd, Denmark). Citric acid monohydrate (Sigma, Saint Louis, MO, United States), sodium phosphate dibasic dehydrate (Riedel-de Haën, Seelze, Germany), and CMC (Sigma, Saint Louis, MO, United States).

### Preparation of Bacterial Cellulose Samples

The BC samples were produced and pretreated according to the methodology previously reported (Han et al., 2018). The Hestrin–Schramm (HS) medium was prepared adding glucose as carbon source (20 g/L), to a mixture of yeast extract and peptone powder as the nitrogen source (5 g/L each). Both were added to distilled water and mixed until dissolution. Afterward the solution was boiled at 100°C for 10 min. The scoby involving bacteria *Acetobacter* (acetic acid bacteria) was added into the HS medium for static cultivation at 26°C for 8 days. The BC samples were then recovered with 3% NaOH solution at 25°C using shaking water bath at 50 rpm for 90 min, neutralized with distilled water adjusted to pH 3.0 using acetic acid for 30 min. In addition, the BC samples were bleached with a 5% H_2_O_2_ solution at 90°C using a shaking water bath at 110 rpm for 60 min. After bleaching, the BC were dried in a drying convection oven (OF-21, Jeio tech Co.) at 35°C (Han et al., 2018).

### Entrapment of Carboxymethyl Cellulose Inside BC

Firstly, BC was swelled with 8% NaOH (v/v) at 25°C for 30 min. Afterward the samples were subjected to ultrasonication, using an ultrasonic bath, at 50°C for 5 min. The BC was neutralized with 0.5% acetic acid (v/v) (Song et al., 2017).

Secondly, different concentrations of CMC (15, 45, and 75 g/L) were dissolved in 20.0 mL of distilled water and the BC samples (1.5 cm^2^) added. The mixtures were placed in a water bath at 55°C for 2, 5, and 24 h. After each period of incubation, the samples were placed in the fridge to stop the reaction, and then BC samples were rinsed repeatedly with distilled water. Finally, the BC samples containing entrapped CMC were freeze-dried (Sadeghi et al., 2012; Lin et al., 2014; Yue et al., 2016).

The entrapment ratio (ER) was determined using Eq. 1, where *W*_*BC*_ is the weight of BC; *W*_*BC+CMC*_ is the weight of the sample after reaction (BC + CMC).

(1) $$\text{ER}\left(\%\right)=\frac{W_{\mathrm{BC}+\mathrm{CMC}}-W_{\text{BC}}}{W_{\text{BC}}}\times 100$$

The results obtained resulted from the mean of three independent experiments.

### *In situ* Laccase-Assisted Synthesis of Polyaniline on BC and BC/CMC

The BC and BC/CMC samples were placed in a 100 mL flask containing a mixture of 25 U/mL of laccase, 5 mM of AOT, 10 mM of KHCF and 50 mM of aniline, in a final volume of 20 mL of 0.1 M acetate buffer (pH 4). The reactional mixtures were covered with foil, allowing the oxygen entrance, and stirred for 24 h at room temperature, in a water bath (Grant, United Kingdom) under shaking. The time of reaction was established according to procedures from literature (Shumakovich et al., 2012; De Salas et al., 2016). At the end of each experiment, the BC samples were washed thoroughly with water to remove the by-products and the remaining starting reagents. The excess of water was removed with filter paper. Afterward the BC samples coated with PANi were dried in a convection oven (OF-21, Jeio tech Co.) at 35°C for 24 h.

### Polymers Characterization

#### UV/Visible Spectroscopy

The polymerization of aniline was followed by UV–Visible spectroscopy using a 96-quartz microplate reader (SynergyMx, Shimadzu, Japan) in the wavelength range of 230–800 nm.

#### ^1^H NMR and MALDI-TOF

The ^1^H NMR spectroscopy of PANi was determined using a Bruker Avance 400 (400 MHz). CDCl_3_ was used as deuterated solvent, using the peak solvent as internal reference. Chloroform was chosen given that it demonstrated the best ability to solubilize most of the reactional content (Shah et al., 2019). The polymer products obtained were also characterized by MALDI-TOF mass spectrometry using a Bruker Autoflex Speed instrument (Bruker Daltonics GmbH) equipped with a 337 nm nitrogen laser. The matrix solution for MALDI-TOF measurements was prepared by dissolving a saturated solution of 2,5-dihydroxybenzonic acid (DHB) in TA30 solution or a saturated solution of α-cyano-4-hydroxycinnamic acid (CHCA). Samples were spotted onto a ground steel target plate (Bruker part *n*° 209519) and analyzed in the linear negative mode by using factory-configured instrument parameters suitable for a 0.4–4 kDa *m/z* range (ion source 1: 19.5 kV; ion source 2: 18.3 kV). The time delay between laser pulse and ion extraction was set to 130 ns, and the laser frequency was 25 Hz. The *M*_*n*_ (number-average molecular weight) and *M*_*w*_ (weight-average molecular weight) of PANi obtained after oxidation was obtained by MALDI-TOF direct analysis and according to the equations:

(2) $$M_{n}=\sum n_{i}\,M_{i}/\sum n_{i}$$

(3) $$M_{w}=\sum n_{i}\,M_{i}^{2}/\sum n_{i}\,M_{i}$$

(4) $$\text{PDI}=M_{w}/M_{n}$$

(5) $${\text{DP}}_{\text{avg}}=M_{n}/\left[\text{M}\right]$$

Where *n*_*i*_ is the relative abundance of each peak; *M*_*i*_ is the *m/z* correspondent to each peak (Su et al., 2017), and [M] is the molecular weight of the repeating unit.

### BC and BC/CMC Surface Coating Characterization

#### FTIR-ATR

To analyze the chemical structure of BC and BC/CMC functionalized with PANi, FTIR-ATR spectra were acquired using an ATR FTIR, IRAffinity-1S (SHIMADZU). 45 scans were completed between 4000 and 400 cm^–1^ at a resolution of 8 cm^–1^. Baselines for each sample spectrum were normalized using spectrum software.

#### X-Ray Diffraction

The changes on the structure of BC and BC/CMC samples when coated with PANi were investigated by X-ray diffraction (XRD) using a New D8-ADVANCE (multi-purpose diffractometer, Bruker-AXS, Fitchburg, United States). X-ray diffraction patterns were recorded at the CuKa radiation wavelength of 1.542 Å and generated at a voltage of 40 kV and a filament emission of 40 mA. Samples were scanned in the range from 5° to 40° at a scan speed of 1/min. The crystallinity index (CI) was calculated from the height ratio between the intensity of the crystalline peak that is sum of crystalline area (*I*_002_ − *I*_*am*_) and the total intensity that means the sum of crystalline and amorphous area (*I*_002_) after subtraction of the background signal measured without BC, following the equation (Lopes et al., 2014; Miyamoto et al., 2014; Mohammadkazemi et al., 2015; Han et al., 2018):

(6) $$\text{CI}=\frac{I_{002}-I_{\text{am}}}{I_{002}}$$

where *I*_200_ is the overall intensity of the peak at 2θ and *I*_*am*_ is the intensity of the base line at 2θ.

#### Thermal Analysis

Thermogravimetric analysis (TGA) measurements of the BC and BC/CMC functionalized samples were carried out in a Perkin Elmer, TGA 4000 equipment, using about approximately 8–10 mg samples over the temperature range, 0–800°C at a heating rate of 10°C/min under a nitrogen flow of 20 mL/min. Thermogravimetric analysis was made with the nanocomposite films placed in a high quality nitrogen (99.5% nitrogen, 0.5% oxygen content) atmosphere to prevent unwanted oxidation. The weight loss calculations were performed considering the main decomposition steps obtained by the DTG curves shown in supporting information (Supplementary Figure S1).

#### Scanning Electron Microscopy

The coated BC and BC/CMC samples were characterized using a desktop scanning electron microscope (SEM) coupled with EDS (energy-dispersive X-ray spectroscopy) detector (Phenom-World BV, Netherlands). All results were acquired using the ProSuite software integrated with Phenom Element Identification software, allowed for the quantification of the concentration of the elements present in the samples, expressed in either weight or atomic concentration. Prior to SEM analysis the samples were added to aluminum pin stubs with electrically conductive carbon adhesive tape (PELCO Tabs^TM^), with the excess removed using compressed air. Samples were coated with 2 nm of Au for improved conductivity. The aluminum pin stub was then placed inside a Phenom Standard Sample Holder, and different points for each sample were analyzed for elemental composition.

#### Color Evaluation by Reflectance Spectrophotometry

The color data for the dried samples was determined using a CCM system (JX-777, Japan) and illuminant D_65_ with a 10° standard observer. The color strength (*K/S*) was calculated from the reflectance values using the Kubelka–Munk equation (Shim and Kim, 2019).

(7) $$K/S={\left(1-R\right)}^{2}/2\,R$$

where *R* is the reflectance, expressed as a proportional value; *K* is the absorption coefficient; and *S* is the light-scattering coefficient. The *K/S* values were presented as checksum *K/S* (sum of all *K/S* values corresponding to all wavelengths). All measurements were performed at least 10 times. The *K/S* values are directly proportional to the sample coloration.

#### Conductivity of BC and BC/CMC Coated Samples

The electrical conductivity of coated samples was measured with a CMT-series (Changmin Tech Co., Ltd.) using four-point probe technique placing them under a pre-defined distance between. The conductivity was calculated according to the following equation:

(8) $$\text{Conductivity}\,\left(\sigma \right)=1/\rho \,\left(\text{S}\,{\text{m}}^{-1}\right)$$

Resistivity can be calculated with ρ = 2π*S*(*V*/*I*), where *S* is the probe spacing (mm), which was kept constant, *I* is the supplied current in microamperes, and the *V* is corresponding voltage measured in millivolts (Yue et al., 2016).

## Results and Discussion

### CMC as Template for Aniline Polymerization

#### Optimization of CMC Entrapment

**FIGURE 1:**
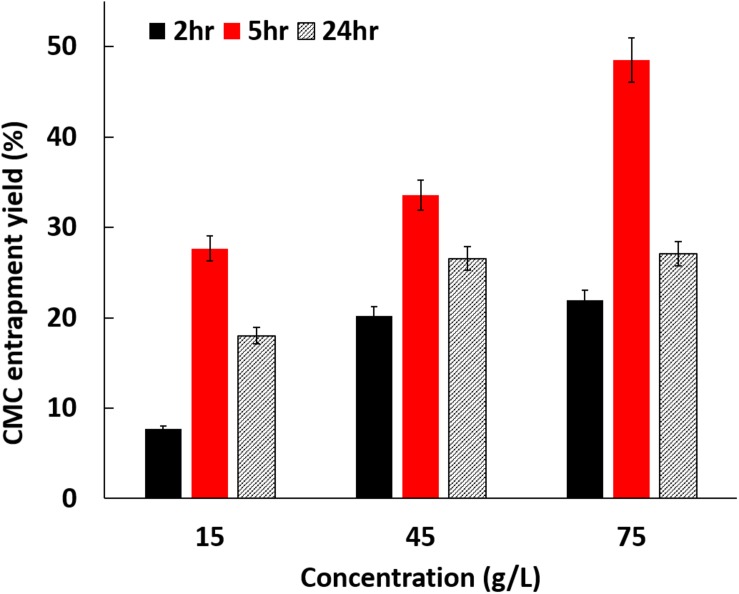
Entrapment yield (%) of CMC inside BC.

Figure 1 shows the entrapment yield of CMC inside BC using different reaction conditions. The data reveal that after 5 h the entrapment of CMC is directly proportional to the concentration used, being achieved close to 50% of material entrapment for 75 g/L of CMC. For the other incubation periods tested (2 and 24 h) the data reveal this proportionality for 15 and 45 g/L, however reaching a saturation plateau for the highest concentration, 75 g/L. The time was also optimized, and the results demonstrate that 5 h is the ideal time to achieve the highest levels of entrapment. Until 5 h the entrapment increases, independently on the initial concentration of CMC, decreasing thereafter for prolonged periods of exposition of BC to CMC. From this data one may assume that from a certain period of time the bonding between CMC and the BC available groups can weaken with the incubation time, due to the high affinity of water to BC (Ballesteros et al., 2018). Regardless this event, one could confirm that, after complex BC/CMC isolation from the medium, no further release of CMC is observed, even during polymerization reaction.

#### The Role of CMC as Template During Aniline Polymerization

The use of laccase for the polymerization of aniline is considered a green methodology comparing to the traditional approaches (Shim et al., 2019a). Su et al. (2018) used laccase from *M. thermophila* for the *in situ* polymerization of aniline into cotton, producing a conductive fabric. A similar approach was described by Zhang et al. (2016) which have used laccase from *Aspergillus* for the functionalization of cotton with PANi.

We previously observed the role of two different additives (AOT and KHCF) on the *in situ* polymerization of aniline in BC (Shim et al., 2019a).

Herein, in order to evaluate the role of CMC as template for aniline polymerization, several laccase-assisted reactions were conducted in the presence of additives and CMC as template. Table 1 presents the percentage of polymer conversion yield increment after aniline polymerization in the presence of CMC and other additives under free solution conditions without BC as support. Comparing the results obtained in the presence of CMC, the polymer conversion is higher for all the reaction conditions tested. The role of the template is less pronounced for samples oxidized only in the presence of laccase whereas when the laccase-assisted reaction was conducted using CMC in the presence of both additives, KHCF and AOT, the polymer conversion reaches the highest increase (14%). We have previously reported that the synthesis of green PANi (emeraldine salt) could by incremented by the inclusion of additives like sodium bis (2-ethyl hexyl) sulfosuccinate (AOT) and KHCF in the medium (Shim et al., 2019a). Considering the previous and the present data one may predict a synergistic effect between both dynamic systems tested (AOT and CMC; Table 1). However, and since the role of AOT and KHCF were previously discussed, only CMC is highlighted herein (Shim et al., 2019a).

**TABLE 1:** Percentage of oligomer/polymer conversion yield increment (%) after polymerization of aniline in presence of CMC under different conditions (calculated from weight of polymer obtained after oxidation and subsequent washing).

Polymerization conditions	Polymer conversion (%)
Aniline + LAC + NO CMC	–
Aniline + LAC + CMC	6
Aniline + LAC + AOT + NO CMC	–
Aniline + LAC + AOT + CMC	9
Aniline + LAC + KHCF + NO CMC	–
Aniline + LAC + KHCF + CMC	10
Aniline + LAC + AOT + KHCF + NO CMC	–
Aniline + LAC + AOT + KHCF + CMC	14

**SCHEME 1:**
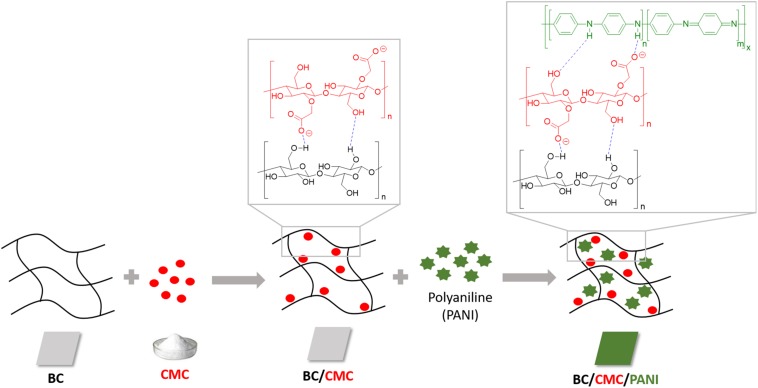
Representation of the non-covalent interactions between BC/CMC/polyaniline during BC/CMC functionalization with polyaniline obtained by laccase oxidation.

These results lead us to the next step of the work by conducting the same experiments using BC as support. In Table 2 is presented the relative yield of polymerization using BC and BC/CMC as support for polymerization, showing the relative percentage of polymer detected, as in the reaction solution or inside the BC network. From the results presented it is perceptible that when CMC is entrapped inside BC and used as polymerization template, the amount of polymer inside the BC network is higher than when the polymerization is conducted using BC without CMC. It seems clear the role of CMC as template for aniline polymerization for the production of oligomers/polymers which remained solubilized or dispersed inside the porous BC membrane without precipitation. The considerable amount of –COO^–^ groups of CMC impart to BC a negative character able to retain higher amount of positive PANi (Lin et al., 2015; Wang et al., 2016; Gautam et al., 2018). Being a macromolecular template, polyanion, able of undergoing electrostatic interactions with PANi, it provides the ideal template for the polymer organization incrementing the amount of polymer entrapped inside BC. The role of CMC as template is distinguishable from the role of an additive since the polymer stays entrapped inside the BC material, even after washing. A scheme representative of the interactions between BC/CMC/PANi is proposed (Scheme 1).

**TABLE 2:** Yield of polymerization of aniline in the presence of additives inside BC and BC/CMC (calculated from weight of polymer obtained after oxidation).

	Control solution without BC	BC (without CMC)		BC/CMC	
		Polymer in BC (%)	Polymer in solution (%)	Polymer in BC (%)	Polymer in solution (%)
Aniline + LAC	100	34	66	54	46
Aniline + AOT + LAC		35	65	60	40
Aniline + AOT + No LAC		–	–	–	–
Aniline + KHCF + LAC		36	64	62	38
Aniline + KHCF + No LAC		14	84	70	30
Aniline + AOT + KHCF + LAC		44	56	53	47
Aniline + AOT + KHCF + No LAC		36	64	62	38

#### Following Enzymatic Polymerization of Aniline by UV–Visible Spectroscopy

**FIGURE 2:**
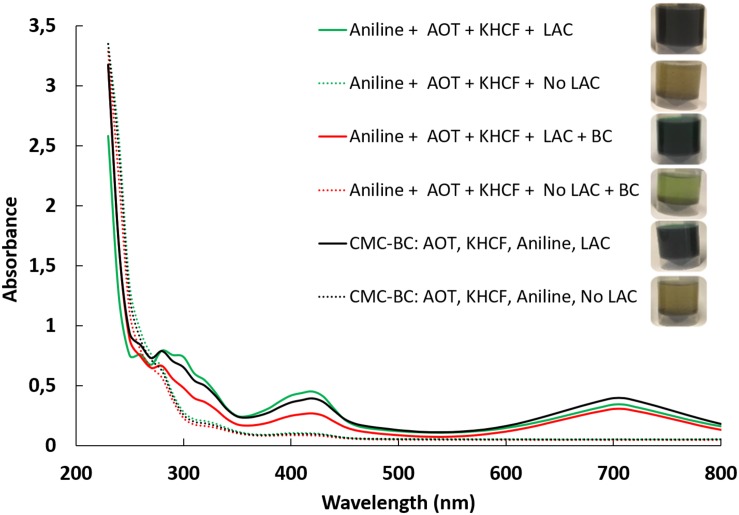
UV–Visible spectra of polyaniline solutions in the presence of template (CMC).

Figure 2 displays the UV–visible spectra of the reaction solutions after aniline polymerization under different conditions. After reaction, the high amount of material entrapped inside the BC fibrils and at their surface, imparts high coloration to BC samples, being that only a low content of soluble oligomers can be detectable by UV/Visible spectroscopy. All the reaction mixtures changed from colorless to dark green during laccase-catalyzed polymerization of aniline in water bath.

The UV–visible spectra of the green PANi synthesized using template/aniline evidences the typical absorption bands of emeraldine salt at 420 nm, distinguishing the semiquinoid radical cation, and at 700 nm, the characteristic signal of doped PANi due to π–semi-polaron electronic transitions (Norris et al., 2000).

The spectra shows a similar profile for reactions conducted in the presence or in the absence of CMC. This might indicate that the type of oligomers/polymers obtained are similar, increasing, however, the amount of polymer entrapped on BC when CMC is used as template. The data gives an indication of the positive role of CMC as potentiating the oxidation leading to the polymerization of higher amount of polymer, as on soluble and/or insoluble form. The data also reveal that the new oligomers are detectable only in the presence of laccase as catalyst.

### Characterization of the Oligomers/Polymers Obtained After Laccase-Assisted Oxidation

#### ^1^H NMR Analysis

**FIGURE 3:**
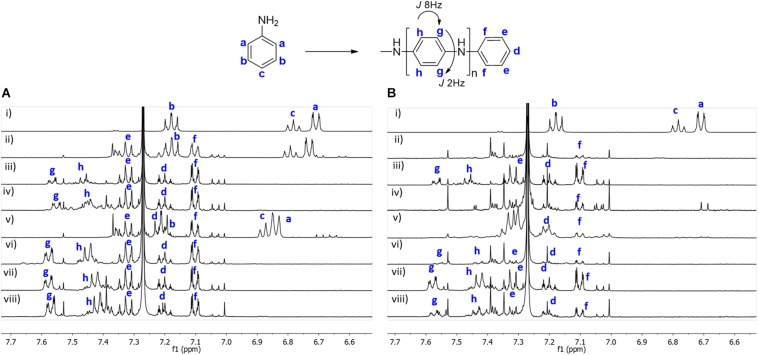
^1^H NMR of polyaniline obtained after *in situ* reaction on BC under different conditions in the absence **(A)** and in the presence of CMC **(B)**. **(A)**: (i) aniline; (ii) aniline + laccase; (iii) aniline + KHCF; (iv) aniline + KHCF + laccase; (v) aniline + AOT; (vi) aniline + AOT + laccase; (vii) aniline + KHCF + AOT; (viii) aniline + KHCF + AOT + laccase; **(B)**: (i) aniline + CMC; (ii) aniline + laccase + CMC; (iii) aniline + KHCF + CMC; (iv) aniline + KHCF + laccase + CMC; (v) aniline + AOT + CMC; (vi) aniline + AOT + laccase + CMC; (vii) aniline + KHCF + AOT + CMC; (viii) aniline + KHCF + AOT + laccase + CMC.

In Figure 3 it is depicted the ^1^H NMR spectra obtained after *in situ* reaction on BC, under different conditions, in the absence (A) and in the presence of CMC (B). The data show that the use of a template to perform the reactions, does not interfere with the structure of the polymer formed. A similar structure is obtained, as in absence or in presence of CMC, as it can be seen by the assignment of the peaks at the same chemical shift and with the same pattern. These results clearly indicate that the catalytic activity and selectivity of laccase is not influenced by the presence of the template. In both cases (BC or BC/CMC), it is observed a set of new doublets (between δ_*H*_ 7.0 and 7.7 ppm), with coupling constants of *J* = 8 Hz and *J* = 2 Hz, which correspond to the *ortho* and *meta* coupling, respectively. These values are in agreement with the PANi structure proposed (Figure 3), which indicates that is *para*-substituted. We have also observed that the polymer conversion using BC is 100% for almost all conditions tested, with exception of aniline + laccase (Figure 3A,ii); or aniline + AOT (Figure 3A,v), evidenced by the peaks **a** and **c,** which appear at a different chemical shift, respecting the control spectra, due to the presence of the polymer in the medium. In the presence of CMC, a full conversion of the aniline monomer is observed for all the conditions tested.

The different oligomeric species of PANi obtained, may have differentiated solubility. By ^1^H NMR, we only observe small oligomeric species, confirmed by the peak’s integration. The results obtained represent only the soluble fraction of the oligomers in the deuterated solvent.

#### MALDI-TOF

**FIGURE 4:**
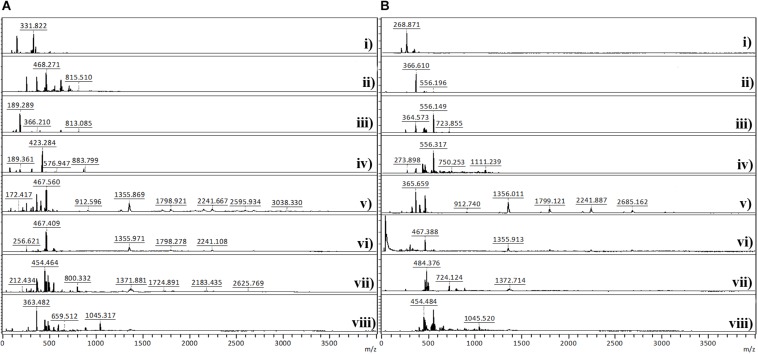
MALDI-TOF analysis of polyaniline obtained after *in situ* reaction on BC under different conditions in the absence **(A)** and in the presence of CMC **(B).** **(A)**: (i) aniline; (ii) aniline + laccase; (iii) aniline + KHCF; (iv) aniline + KHCF + laccase; (v) aniline + AOT; (vi) aniline + AOT + laccase; (vii) aniline + KHCF + AOT; (viii) aniline + KHCF + AOT + laccase; **(B)**: (i) aniline + CMC; (ii) aniline + laccase + CMC; (iii) aniline + KHCF + CMC; (iv) aniline + KHCF + laccase + CMC; (v) aniline + AOT + CMC; (vi) aniline + AOT + laccase + CMC; (vii) aniline + KHCF + AOT + CMC; (viii) aniline + KHCF + AOT + laccase + CMC.

The MALDI-TOF analysis was conducted to determine the degree of polymerization of PANi. Figure 4 shows the MALDI-TOF spectra of PANi produced after *in situ* reaction on BC under different conditions in the absence and in the presence of CMC. The mass spectra of BC/PANi shows peaks between 189 and 3038 *m/z*, whereas the spectra of BC/CMC/PANi display peaks between 268 and 2685 *m/z*. Considering the monomer unit of PANi (C_6_NH_5_) = 93, the peaks found might be attributed to aniline dimers, trimers, and tetramers. Looking to both spectra, it can be seen that longer polymers are detected on the reaction solution, in the absence of CMC. When this compound is used as template, the longer oligomers/polymers might be entrapped inside BC and not accessible for detection.

Table 3 displays the characterization of the oligomers/polymers after oxidation and isolation, calculated from MALDI-TOF data analysis. The data confirms the occurrence of oligomers and polymers after aniline laccase-assisted polymerization since low and high molecular mass polymeric fractions were detected. We have detected oligomerization degrees of up to 33 residues in the PANi obtained in the presence of AOT and laccase and up to nine residues in the soluble green products obtained in the presence of KHCF. However, the average DP obtained corresponded to oligomers with a maximum of 13 units of aniline. These results have been found previously by us when conducting the experiments in the presence of additives (KHCF and AOT) in the absence of template (Shim et al., 2019a). Herein, our goal was to infer if the use of a template like CMC would influence the degree of polymerization. From the data obtained it seems that it is not the case, similar DPs are achieved when the experiments are conducted in the presence of CMC as template. However, data from Table 2 and other results like stronger coloration and conductivity, lead us to assume that higher amount of polymer is entrapped inside BC which, due to mobility constrains, are not released to the reaction solution, remaining inside the BC network. A higher amount of conductive polymer inside the porous support material is expected to confer greater electric conductivity.

**TABLE 3:** Polyaniline characterization in terms of *Mn*, *Mw*, average polymerization degree (DP_*avg*_) and maximum degree of polymerization (DP_*max*_) after laccase oxidation on BC and on BC/CMC under different conditions (the data is obtained from MALDI-TOF analysis; aniline (M*w*) = 93,13 g/mol).

	*M*_*n*_		*M*_*w*_		PDI (*M_*w*_/M_*n*_*)		DP_*avg*_		DP_*max*_	
	BC	BC/MC	BC	BC/CMC	BC	BC/CMC	BC	BC/CMC	BC	BC/CMC
Aniline + LAC	477	384	530	391	1.1	1.0	5	4	10	7
Aniline + KHCF + LAC	593	485	633	517	1.1	1.1	6	5	9	9
Aniline + KHCF + No LAC	645	656	767	764	1.2	1.2	7	7	15	12
Aniline + AOT + LAC	1092	1114	1808	1753	1.7	1.6	12	12	33	31
Aniline + AOT + No LAC	876	1172	1470	1805	1.7	1.5	10	13	29	29
Aniline + KHCF + AOT + LAC	702	659	1123	803	1.6	1.2	8	7	29	15
Aniline + KHCF + AOT + No LAC	599	657	718	755	1.2	1.1	7	7	15	15

*The values were obtained using the equations previously detailed in the methods section.*

#### FTIR

FTIR-ATR spectra for BC and BC/CMC functionalized with PANi under different conditions are presented in Supplementary Figure S2. The BC spectra contains all the peaks characteristic of cellulose (Jasim et al., 2017), showing the typical peak of the intramolecular hydrogen bond at 3340 cm^–1^, and the C–O stretching vibration region at 1045 cm^–1^ (Feng et al., 2012). After entrapment of CMC inside cellulose nanofibers, the band at 3348 cm^–1^ became broader, as can be evidenced by the BC/CMC spectrum (Supplementary Figure S2; Basavaraja et al., 2013). This spectrum shows strong absorption bands at 3348, 2931 and 1059 cm^–1^ corresponding to the characteristic O–H stretching vibrations of CMC (Pushpamalar et al., 2006; Fu et al., 2015; Kotresh et al., 2016). The absorption peak of PANi at 1565 cm^–1^ due to C=C stretching deformation of quinoid rings stretching deformations, can be detected (Supplementary Figure S2); Peng et al., 2012; Kotresh et al., 2016). The spectra of PANi obtained without laccase (b3) and with laccase (b2) reveal a similar profile, indicating that PANi is produced even without catalyst in the presence of molecular oxygen. Moreover, these findings have been previously demonstrated through UV/Vis spectra. The PANi formed in the absence of laccase resulting from oxidation is not easily detectable by UV/Vis spectra since this technique is only able the measure soluble samples. However, from the images supplied in Figure 2, it is possible to detect coloration of the samples even in the absence of the catalysts, which confirms our findings. The presence of the additives played herein a crucial role, as previously confirmed by De Salas et al. (2016) that observed the aniline oxidation only in the presence of both AOT and KHCF additives, confirming the complete or partial doping. BC/PANi have equal quinoid and benzoid units, as indicated by their similar band intensities, owning a conductive-like structure which would confer to BC or BC/CMC functionalized materials conductive properties.

#### X-Ray Diffraction

The XRD spectra of the extended linear scanning (10–60°) of BC and BC/CMC functionalized with PANi is shown in Supplementary Figure S3. This technique will give some information concerning the structural changes in the microstructure of BC. Crystallinity and strength of hydrogen bonding accounts in the microstructure of this material. The XRD spectrum of BC show peaks at 2θ = 22.9–23.2°, the typical profile of cellulose I, assigned to the atomic plane of [2 0 0] of cellulose crystallite (Bhadra et al., 2009; Feng et al., 2012; Yousefi et al., 2013). When BC is modified with CMC, this peak appears more clearly and prominently, while BC/PANi composite showed an additional peak at 2θ = 11.5°. This peak results from a decrease in the extent of ordered cellulose due to a weak acidic degradation (Jasim et al., 2017). In addition, the peak of polymer composites detected around 2θ = 32.8°and 34.9° (Supplementary Figure S3, a2 and b2) corresponding to diffractions of (3 0 0) and (2 0 2), respectively (Wan et al., 2006). The crystallite size is estimated from the half width for the (2 0 0) diffraction as previously described (Yamamoto et al., 1996). From the results obtained one can observe that when BC and BC/CMC were functionalized with oligomer/polymers, the composites present higher crystallinity and crystallite size (Supplementary Tables S1, S2). Crystallinity of BC/CMC materials is, however, lower than BC. The decrease of crystallinity upon CMC entrapment is associated to the split of the broadening hydrogen bonds resulting from the carboxymethyl replacement at the hydroxyl groups of cellulose (Casaburi et al., 2018). The CMC remains between the cellulose ribbons hindered their approaching and preventing crystallization (Chen et al., 2014). It is assumed that the carboxymethylated BC lead to the increase of the amorphous parts, which also offers to BC/CMC higher adsorption capacity (Chen et al., 2009). In the case of BC and BC/CMC functionalized with PANi, the crystallinity increases, as the polymer chains ordering and regularity increases. The increase of the crystallinity might be attributed to the interaction of the PANi with the -OH groups of BC or CMC, resulting in a uniform distribution of the polymer (Supplementary Table S1).

#### Thermal Degradation Stability

**FIGURE 5:**
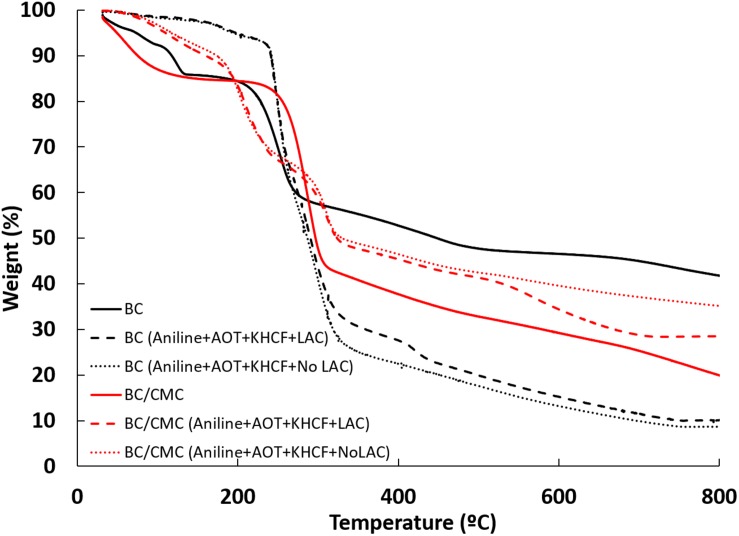
TGA analysis of BC and BC/CMC materials functionalized with polyaniline under different conditions.

The TGA curves of BC composites are shown in Figure 5. Regarding the controls, BC and BC/CMC have a similar thermal degradation until 300°C, losing near 45% of weight. At 800°C, the complex loses almost all the initial weight (80%), whereas BC loses 60% of the weight. BC structure may be more robust than when CMC is entrapped, due to the release of water content when the entrapment occurs. Therefore, this event could lead to a change in the packing of the cellulose chains of BC, inducing some loss of its thermostability.

In all the cases presented, the first degradation stage is related to the presence of moisture in the samples (Müller et al., 2012) and the second stage of weight loss occurring at 300°C is due to the thermal-oxidative degradation of the PANi structure and loss of small molecules (Gautam et al., 2018). When the materials are functionalized with PANi, the thermal behavior of BC and BC/CMC differs. BC/CMC functionalized with PANi present higher stability from up to 300°C (red dot and dash lines) than comparing to the respective control (red full line), attributed to the integrated compact morphology of the composites. A stronger interaction among all components of the materials, could led to a higher thermostability. As observed when CMC is entrapped inside BC (comparing black line with red line), when PANi is inside BC (black dot and dash lines) the packing of the cellulose chains can be more disorganized, leading to lower thermostability.

In Table 4 are presented in detail the thermal events occurred during TGA analysis, namely the decomposition temperatures (T_*dmax*_) and respective weight loss (Δweight) of the BC and BC/CMC functionalized with PANi.

**TABLE 4:** Decomposition temperatures (T_*dmax*_) and respective weight loss (Δweight) of the BC and BC/CMC functionalized with PANi (the weight loss calculations were performed considering the main decomposition steps obtained by the DTG curves shown in supporting information – Supplementary Figure S1).

BC						BC/CMC					
–		Aniline		Aniline		–		Aniline		Aniline	
		KHCF + AOT + LAC		KHCF + AOT + No LAC				KHCF + AOT + LAC		KHCF + AOT + No LAC	
					
Δ weight (%)	T_*dmax*_ (°C)	Δ weight (%)	T_*dmax*_ (°C)	Δ weight (%)	T_*dmax*_ (°C)	Δ weight (%)	T_*dmax*_ (°C)	Δ weight (%)	T_*dmax*_ (°C)	Δ weight (%)	T_*dmax*_ (°C)
3	44	6	129	6	186	14	62	10	117	9	109
3	84	31	248	37	251	42	289	23	211	24	203
7	123	32	293	31	299	22	568	19	311	18	310
29	251	9	420	16	580	–	–	6	432	7	423
10	451	12	636	–	–	–	–	13	574	7	657
5	728	–	–	–	–	–	–	–	–	–	–

#### Surface Morphology

**FIGURE 6:**
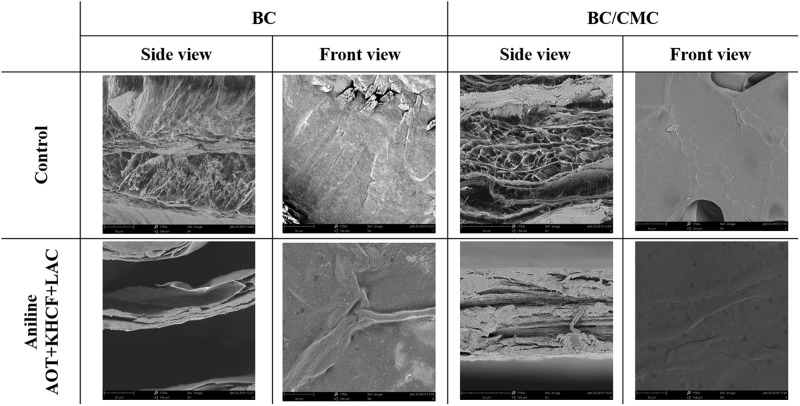
SEM photographs of BC and BC/CMC functionalized with PANi in the presence of laccase and additives (×1750 magnification).

SEM micrographs of BC and BC/CMC functionalized with PANi in the presence of laccase and additives are presented in Figure 6. The surface of all samples discloses the characteristic highly porous three-dimensional network of cellulose, responsible for the excellent mechanical properties of the biopolymer, as well as for the high water absorption capacity and liquid retention.

Though the crosslinking density of the cellulosic network has become denser in the BC/CMC composites, they did not exhibit a remarkable change in the nanofibers morphology in what concern length and thickness comparing to neat BC membranes. Moreover, CMC incorporation into BC nanofibers led to the reduction of the composites’ porosity as a simple result of the diffusion of CMC in the BC membrane.

The surface of BC/CMC by front view reveals a smoother and more compact structure than BC. The higher conversion capacity of BC/CMC may have resulted from the wider dimension of fibrils and larger pore dimension of the network, which provided more spaces for PANi deposition and incremented the area available for enzyme-catalyzed reactions (Fu et al., 2015).

#### Electrical Conductivity and Coloration of BC and BC/CMC Functionalized With PANi

**FIGURE 7:**
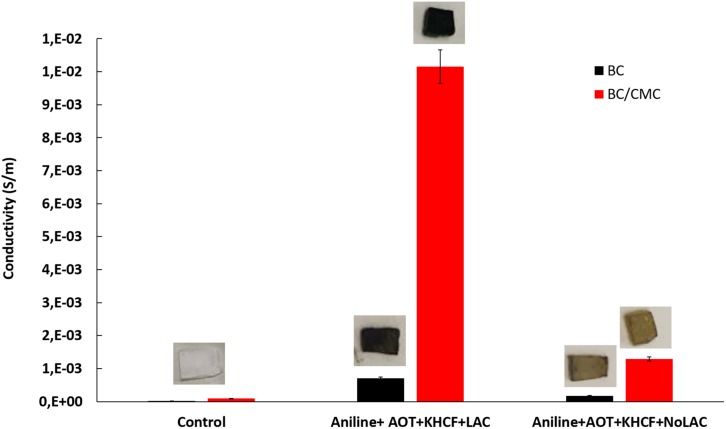
Conductivity of BC and BC/CMC functionalized with polyaniline oxidized by laccase in the presence of additives, AOT and KHCF; images of the samples are presented in the upper part of each graph bar.

Figure 7 shows the electrical conductivity and color evaluation of BC and BC/CMC functionalized with PANi. The conductivity of BC/CMC/PANi is considerable higher than that of BC/PANi. The CMC template, as mentioned previously, allowed the deposition of higher amount of oligomers/polymers inside the BC network which conferred not only higher electrical conductivity but also incremented coloration. The negatively charged carboxylate groups of CMC react by ionic interactions with the protonated amino (–NH_2_) groups of PANi to form biopolymer complex (Peng et al., 2015; Gautam et al., 2018). Electrical conductivity of PANi depends essentially on the degree of polymerization, oxidation state, particle morphology, crystallinity, interior intra-chain interactions and molecular weight. These properties were altered by CMC addition conferring to the composites 10 times higher electrical conductivity (Jasim et al., 2017). Comparing with the previous data reported by Shim et al. (2019a), the use of CMC on the system allowed to increment the conductivity results from 2E^–04^ to 1E^–02^, giving a strong evidence of the role of the template on promoting aniline oxidation and production of highly conductive materials.

The images of colored samples confirm the direct correlation between the amount of polymer entrapped inside BC and the conductivity. The higher is the degree of polymerization and amount of polymer entrapped the higher is the color strength (checksum *K/S*; Table 5).

**TABLE 5:** Color evaluation of BC and BC/CMC samples after aniline oxidation by laccase.

		*K/S* (Checksum)
BC	–	1.99
	Aniline + AOT + KHCF + LAC	282.32
	Aniline + AOT + KHCF + No LAC	88.06
BC/CMC	–	0.61
	Aniline + AOT + KHCF + LAC	341.83
	Aniline + AOT + KHCF + No LAC	74.94

It is noteworthy that the role of laccase as catalyst in the polymerization of PANi is more evident at this stage. Despite the similarity observed between the oligomers of the soluble phase, obtained in the absence or in the presence of laccase, the data herein presented highlighted the role of laccase as an effective catalysts of aniline polymerization. The oligomers synthetized by laccase, due to the template-assisted reaction, are more retained inside BC/CMC than the oligomers produced in its absence, increasing exponentially the coloration of the materials and their conductive behavior (Figure 7 and Table 5).

## Conclusion

Herein we study the role of CMC as a template for the enzymatic polymerization of aniline inside BC membranes. The best CMC entrapment conditions were established according to the highest entrapment yield obtained (75 g/L CMC, 1.5 cm^2^ BC, 5 h, 55°C). The oxidation experiments conducted using BC and BC/CMC as supports for aniline polymerization in the presence of additives revealed that the type of oligomers obtained is the same, independently on the use of a template (CMC). It is, however, noteworthy that the presence of this template increments the amount of oligomers entrapped inside BC, as adsorbed or bond to the network structure. The template promotes the fixation of higher amount of PANi to BC porous structure, incrementing the electrical conductivity and coloration of the materials. The best conditions to produce electrically conductive BC/CMC were found to be: 25 U/mL of laccase, 5 mM of AOT, 10 mM of KHCF, 50 mM of aniline, in acetate buffer (pH 4), for 24 h at 35°C.

## Data Availability Statement

All datasets generated for this study are included in the article/Supplementary Material.

## Author Contributions

ES was responsible for experimental and writing details. JN was responsible for HNMR data curation. AC-P was the co-supervisor of the work. CS was responsible for experimental details and manuscript revision. HK was the supervisor of the work. All authors have read and approved the final version of the manuscript.

## Conflict of Interest

The authors declare that the research was conducted in the absence of any commercial or financial relationships that could be construed as a potential conflict of interest.
