# Supplementary material for: Carboxymethyl Cellulose (CMC) as a Template for Laccase-Assisted Oxidation of Aniline
Source: Front Bioeng Biotechnol. 2020 May 14;8:438. doi: 10.3389/fbioe.2020.00438 (PMC7240045; doi:10.3389/fbioe.2020.00438)
Supplement: Supplementary file 1 [file Data_Sheet_1.pdf]

Supporting Information

Carboxymethyl cellulose (CMC) as a template for laccase-assisted oxidation of aniline

Euijin Shim[1], Jennifer Noro[3], Artur Cavaco-Paulo[2,3], Hye Rim Kim[1]*, Carla Silva[3]*

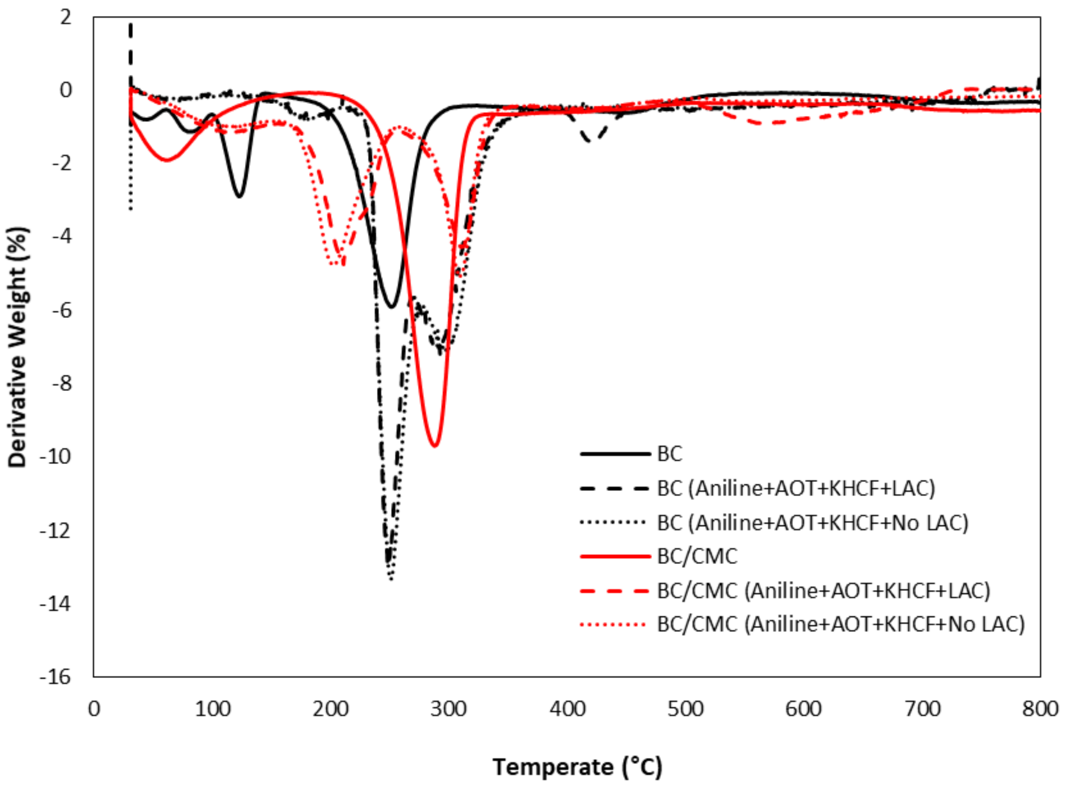

Figure S1: DTGA curves of BC and BC/CMC materials functionalized with polyaniline under different conditions.

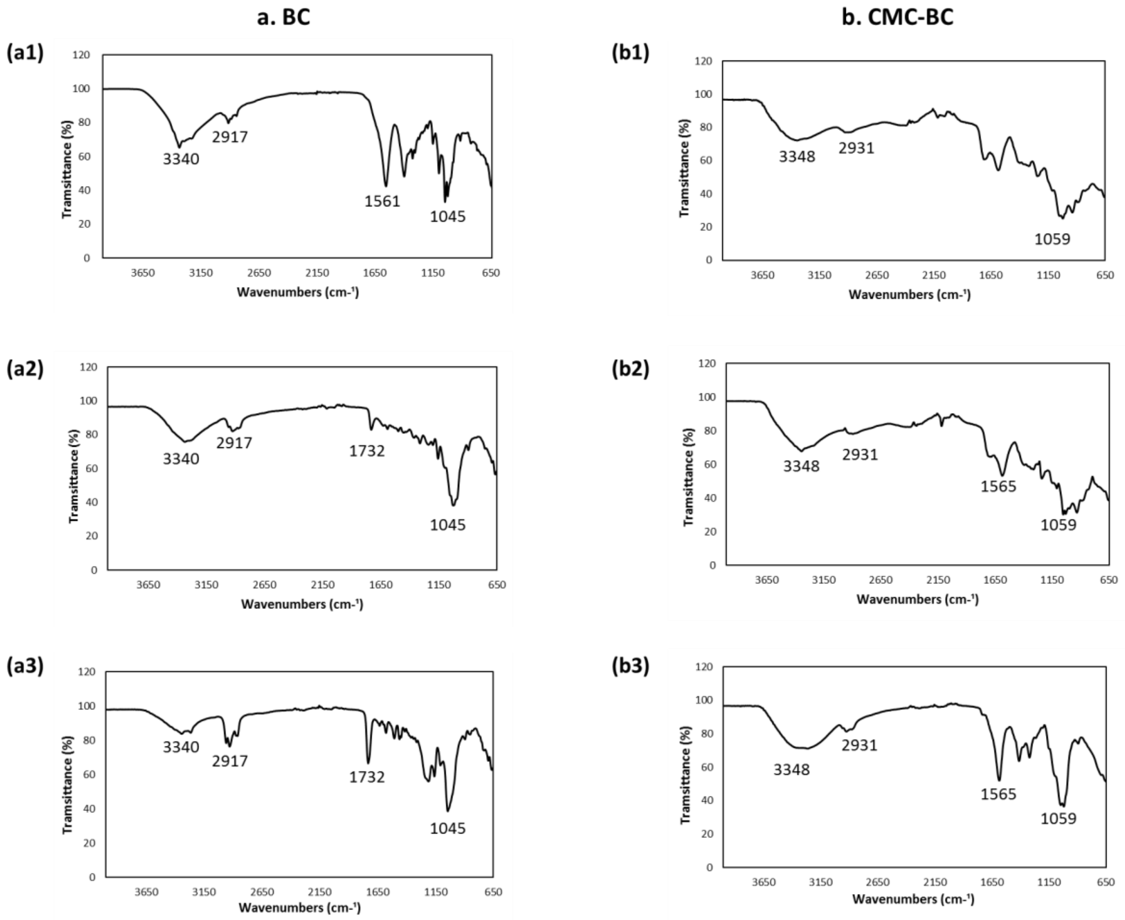

Figure S2: FTIR-ATR of BC and BC/CMC samples functionalized with polyaniline: (a1) BC; (a2) BC (aniline+AOT+KHCF+LAC); (a3) BC (aniline+AOT+KHCF+No LAC); (b1) BC/CMC; (b2) BC/CMC (aniline+AOT+KHCF+LAC); (b3) BC/CMC (aniline+AOT+KHCF+No LAC).

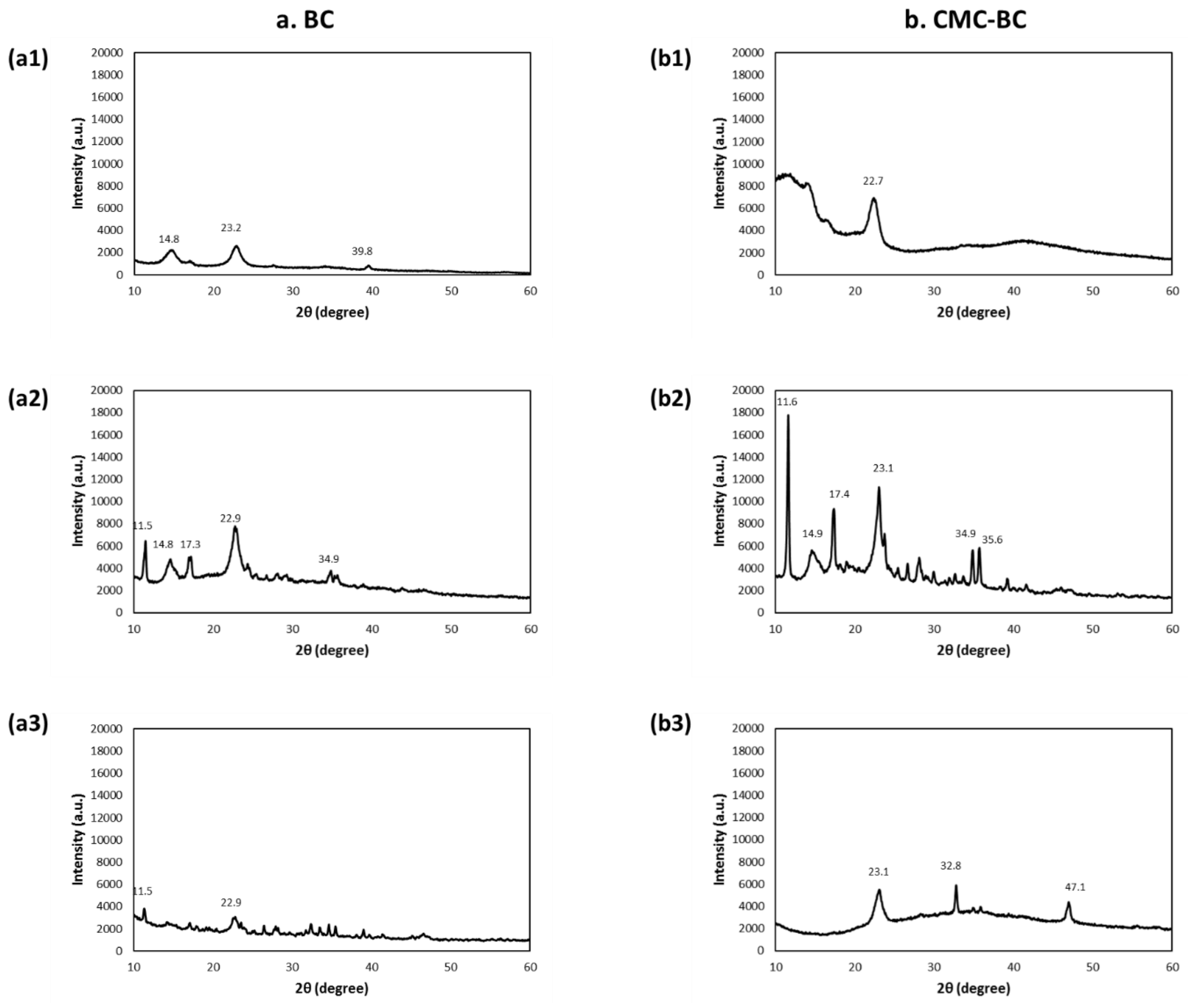

Figure S3. X-ray diffraction analysis of BC and BC/CMC samples functionalized with polyaniline: (a1) BC; (a2) BC (aniline+AOT+KHCF+LAC); (a3) BC (aniline+AOT+KHCF+No LAC); (b1) BC/CMC; (b2) BC/CMC (aniline+AOT+KHCF+LAC); (b3) BC/CMC (aniline+AOT+KHCF+No LAC).

Table S1. Cellulose crystallite size of BC/PANi and BC/CMC/PANi samples

	2θ (°)	Crystallite size (Å)
**BC**	14.8	67.8
	23.2	68.3
**BC (aniline + KHCF + AOT + LAC)**	11.5	279.1
	14.8	86.5
	17.3	170.7
	22.9	87.7
	34.9	107.1
**BC (aniline + KHCF +AOT + No LAC)**	11.5	62.7
	22.9	80.5
**BC/CMC**	22.7	73.2
**BC/CMC (aniline + KHCF + AOT + LAC)**	13.6	364.2
	14.9	73.5
	17.4	243.5
	23.1	128.7
	34.9	335.5
	35.6	339.5
**BC/ CMC (aniline + KHCF +AOT + No LAC)**	23.3	82.6
	32.8	333.1

Table S2. Cellulose crystallinity of BC/PANi and BC/CMC/PANi samples

	Crystallinity (%)
**BC**	69.3
**BC (aniline + KHCF + AOT + LAC)**	76.2
**BC (aniline + KHCF +AOT + No LAC)**	71.7
**BC/CMC**	56.4
**BC/CMC (aniline + KHCF + AOT + LAC)**	70.3
**BC/ CMC (aniline + KHCF +AOT + No LAC)**	62.7
